# Metabolic and Endocrine Characteristics of Indian Women with Polycystic Ovary Syndrome

**DOI:** 10.22074/ijfs.2016.4764

**Published:** 2016-04-05

**Authors:** Amar Nagesh Kumar, Jupalle Nagaiah Naidu, Uppala Satyanarayana, Krishnan Ramalingam, Medabalmi Anitha

**Affiliations:** 1 Department of Biochemistry, Narayana Medical College and Hospital, Nellore, Andhra Pradesh, India; 2 Department of Biochemistry, Dr Pinnamaneni Siddartha Institute of Medical Sciences, Gannavaram, Andhra Pradesh, India; 3 Department of Obstetrics and Gynecology, Narayana Medical College and Hospital, Nellore, Andhra Pradesh, India

**Keywords:** Polycystic Ovary Syndrome, Gonadotropin Hormones, Insulin Resistance, Dyslipidemia, Hypothyroidism

## Abstract

**Background:**

Polycystic ovary syndrome (PCOS) is one of the most common endocrinological disorders among women of reproductive age and the leading cause of female infertility. This study intends to evaluate the lipid profile, hormonal levels [free T3 (fT3), free T4 (fT4), thyroid stimulating hormone (TSH), insulin, luteinizing hormone (LH), follicle stimulating hormone (FSH), and prolactin] in PCOS women from Nellore and its surrounding districts of Andhra Pradesh, India.

**Materials and Methods:**

This cross-sectional study included 80 newly diagnosed PCOS women and an equal number of age and body mass index (BMI) matched healthy controls. We used the photometry methods to determine serum glucose levels and the lipid profile. An immunoturbidometry method was employed to measure high sensitive C-reactive protein (hsCRP). All hormonal parameters were measured using chemiluminescence immunoassays. Insulin resistance was evaluated using the homeostatic model assessment-insulin resistance (HOMA-IR) method. Statistical analysis was done using SPSS software version 20.0.

**Results:**

The PCOS patients presented statistically higher levels of total cholesterol (TC), triglycerides (TG) and low density lipoprotein cholesterol (LDL-c, P<0.0001) when compared to those of controls. PCOS patients had elevated fasting glucose, hsCRP, fasting insulin, TSH, LH and prolactin levels (P<0.001). An increased LH/FSH ratio (>1.5) was seen in women with PCOS compared with control women. In addition, we observed a direct correlation between fasting insulin with fasting glucose and HOMA-IR. LH was inversely proportional to BMI.

**Conclusion:**

The present study showed a higher prevalence of insulin resistance, dyslipidemia, and hypothyroidism in PCOS women. Furthermore this study showed increased LH concentrations, a higher LH/FSH ratio, and higher prolactin levels in PCOS women.

## Introduction

Polycystic ovary syndrome (PCOS) is one of the most common endocrinological disorders among adolescent girls and women of reproductive age. PCOS is the leading cause of female infertility (1). Menstrual irregularity, chronic anovulation, hyperandrogenism, and multiple small sub-capsular cystic follicles in the ovary on ultrasonography characterize the syndrome. PCOS is associated with insulin resistance, increased risk of type 2 diabetes mellitus and cardiovascular disorders (2). Obesity, mainly central obesity, is present in varying degrees (30-70%) in women with PCOS (3, 4). Central obesity, being a prominent feature of the so-called metabolic syndrome, is directly linked to increased peripheral insulin resistance (5). It has been shown that insulin resistance is responsible for the development of polycystic ovaries in PCOS women although obesity seems to be the major cause (6). Hence pathogenic determinants of PCOS include insulin resistance and β-cell dysfunction. Therefore, women with PCOS have an increased risk for type 2 diabetes (7).

The majority of studies that evaluated the prevalence of glucose intolerance in PCOS primarily included obese women, which aggravated their risk for glucose intolerance. Likewise, a high prevalence of abnormal glucose intolerance has also been documented in women with PCOS (6). An elevated luteinizing hormone/follicle stimulating hormone (LH/FSH) ratio is typically seen in PCOS patients. This elevated ratio was considered as a gold standard for clinical diagnosis of the disease (8) before the proposal of the Rotterdam criteria. However LH/FSH levels, as a gold standard, became controversial after a number of studies have reported a variable prevalence of these ratios (30-90%) among PCOS women (9, 10). An ethnic variation of the metabolic and endocrine pattern in PCOS was also reported (11-13).

All features of this syndrome may not be present in an individual patient (2, 13). Depending on the interactions of different hormones in PCOS patients, the pathogenesis, clinical presentation, and biochemical profile varies in an individual. In general, the treatment of PCOS patients is targeted towards regularization of menses and recover of fertility. PCOS women are at high risk of developing type 2 diabetes, cardiovascular disorders, and endometrial carcinoma (2, 7, 9). Hence, in addition to symptomatic relief, correction of the underlying endocrinological pathology and biochemical abnormalities at the earliest is necessary. Hence biochemical parameters and the hormone profile become important in understanding the pathogenesis of PCOS. Assessment of the lipid profile, glycemic status and endocrine status in PCOS patients may help in making a decision regarding treatment, better outcome, differential diagnosis, and prognosis of the disease. With this background we have planned the present study to assess the metabolic profile and endocrine pattern of PCOS women from Nellore and its surrounding districts of Andhra Pradesh, India.

## Materials and Methods

We conducted this cross-sectional study at Narayana Medical College and Hospital, Nellore, Andhra Pradesh, India during the period of October 2012 to January 2014. The study comprised 80 newly diagnosed PCOS women and an equal number of age and body mass index (BMI) matched healthy females as controls. All participants were in the age group of 19 to 35 years. Patients have been diagnosed with PCOS on the basis of the Rotterdam criteria (14). A total of two out of three of the following are required for diagnosis: oligoand/ or anovulation (defined by the presence of oligomenorrhea or amenorrhea); clinical and/or biochemical signs of hyperandrogenism [defined by presence of hirsutism (Ferriman-Gallwey score ≥6), acne or alopecia, and/or elevated androgen levels]; and polycystic ovaries by gynecological ultrasound. We excluded patients with congenital adrenal hyperplasia, Cushing’s syndrome, androgen- secreting tumors, known hypothyroidism on treatment and intake of any medication that affected endocrinal parameters.

Height and weight were obtained from each subject. The BMI was calculated as the weight in kilograms divided by the square of height in meters. About 5 ml of blood was collected from the antecubital vein. Fasting blood samples were collected in plain and sodium fluoride tubes, and then centrifuged at 3500 rpm for 10 minutes to separate the serum. Analysis of total cholesterol (TC), triglycerides (TG), high density lipoprotein cholesterol (HDL-c) and glucose were performed using commercial kits available for a fully automated Humastar 600 biochemistry analyzer (Germany). We used Friedwald’s formula to calculate low density lipoprotein cholesterol (LDL-c) and very low density lipoprotein (VLDL-c) levels. High sensitive C-reactive protein (hsCRP) levels were measured by immunoturbidometry (hsCRP Reagent Kit, CRP-ULTRA turbilatex, Spinreact, Spain). Hormones free T3 (fT3), free T4 (fT4), thyroid stimulating hormone (TSH), LH, FSH, prolactin and insulin were measured by the chemiluminescence immunoassay (CLIA) method using a Beckman Coulter Access fully automated analyzer. The hormone kits used in the Beckman Coulter Access analyzer (USA) were from Beckman Coulter, Ireland. We estimated insulin resistance by the homeostatic model assessment-insulin resistance (HOMAIR) method (15).

### Statistical analysis

All the results were tabulated as mean and standard deviation. We used the SPSS 20.0 version for statistical analysis. The unpaired student t test was used to determine the statistical significance between the study groups. Pearson correlation was used for correlating different parameters. A P value of ˂0.05 was considered to be statistically significant.

### Ethical considerations

The study was approved by the Narayana Medical College and Hospital Institutional Ethics Committee, Nellore, Andhra Pradesh, India. Written and informed consent was obtained from the individuals who have participated in the study.

## Results

There were 80 clinically proved, confirmed PCOS patients in the age range of 19-35 years chosen for the study. The mean age of controls was 26.7 ± 3.4 years and for PCOS patients, it was 25.6 ± 3.9 years (P=0.06). The mean BMI for controls was 26.1 ± 4.2 kg/m^2^ and 27.0 ± 5.6 kg/m^2^ for PCOS patients (P=0.25). There was no significant statistical difference in age or BMI between PCOS women and controls. We assessed both PCOS women and controls for serum lipid profile, fasting blood sugar, fasting insulin, insulin resistance (HOMA-IR), thyroid profile, LH, FSH, LH/FSH ratio and prolactin levels. Comparisons were made between the two groups. The results of biochemical and hormonal profile findings of this study are shown in Table 1.

**Fig 1:**
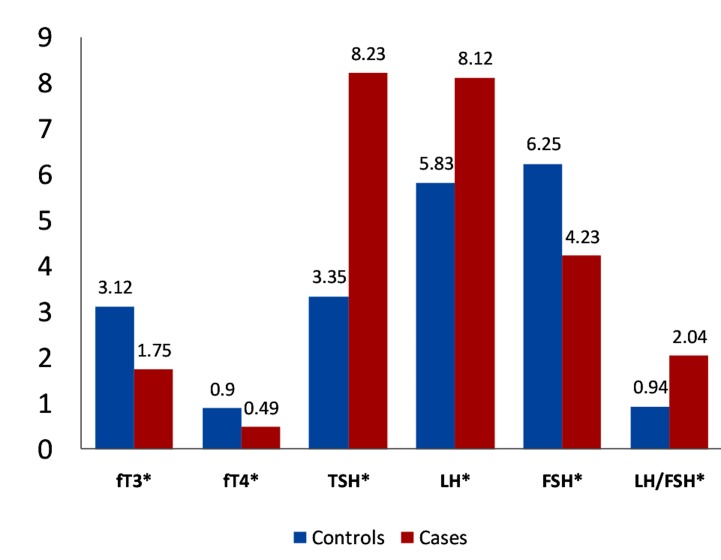
Mean values for gonadotropin hormones in control women and polycystic ovary syndrome (PCOS) women (cases). *; P<0.0001, fT3; Free T3, fT4; Free T4, TSH; Thyroid stimulating hormone, LH; Luteinizing hormone and FSH; Follicle stimulating hormone.

As seen in Table 1, PCOS patients had higher TC, TG, LDL-c and VLDL-c levels (P˂0.001) compared to controls which indicated that PCOS women had dyslipidemia. On the other hand, HDL-c showed no significant statistical difference (P=0.481) between the groups. Fasting glucose, fasting insulin and insulin resistance showed a significant increase (P˂0.001) in cases compared to controls. Figure 1 shows a graphical representation of the mean values of the thyroid and gonadotropin hormones. PCOS patients had increased TSH compared to controls (P<0.0001). LH and prolactin showed increase levels (P<0.0001) whereas FSH (P<0.0001) showed mildly decreased levels in PCOS women compared to healthy controls (Table 1).

Significant positive correlations between BMI with fasting insulin (r=0.493) and insulin resistance (r=0.401, P˂0.01) and a significant negative correlation with LH (r=-0.279, P˂0.01) were found in PCOS patients (Table 2). hsCRP positively correlated with fasting glucose (r=0.816), fasting insulin (r=0.518), insulin resistance (r=0.609), LH/FSH ratio (r=0.631) and prolactin (r=0.688, P<0.01), and had a negative correlation with FSH (r=-0.514, P<0.001). Fasting glucose showed positive correlations with fasting insulin (r=0.703), insulin resistance (r=0.811), LH/FSH ratio (r=0.615), and prolactin (r=0.699, P<0.01), and a negative correlation with FSH (r=-0.533, P<0.01). Similarly, fasting insulin also showed significant positive correlations with insulin resistance (r=0.981) and prolactin (r=0.542, P<0.01). TSH showed positive correlations with BMI, hsCRP, fasting glucose, fasting insulin, insulin resistance, LH/FSH ratio and prolactin (r=0.173, P<0.05, r=0.757, r=0.772, r=0.499, r=0.583, r=0.492, r=0.693, respectively and P<0.01). LH showed a positive correlation with LH/FSH ratio (r=0.556, P<0.01). FSH showed a negative correlation with LH/FSH ratio (r=-0.571, P<0.01). The LH/ FSH ratio showed a positive correlation with prolactin (r=0.469, P<0.01).

**Table 1:** Serum concentrations of lipid profiles and hormones in normal controls and polycystic ovary syndrome (PCOS) women


Parameters and their normal ranges	Controls (Mean ± SD)	PCOS cases (Mean ± SD)	P value

TC (<200 mg/dl)	181 ± 32.9	214 ± 35.7	0.0001
TG (60-165 mg/dl)	105 ± 48.9	189 ± 42.9	0.0001
HDL-c (45-65 mg/dl)	47.5 ± 8.5	46.6 ± 7.6	0.481
LDL-c (<130 mg/dl)	114 ± 20.1	130 ± 30.2	0.001
VLDL-c (12-40 mg/dl)	21 ± 9.8	37.3 ± 8.8	0.001
Fasting glucose (70-110 mg/dl)	86.0 ± 9.1	127 ± 11.4	0.0001
Fasting insulin (0.7-9.0 μlU/ml)	7.4 ± 1.8	13.4 ± 5.3	0.001
HOMA-IR (<2.0)	1.6 ± 0.5	4.3 ± 2.0	0.001
fT3 (2.50-3.90 pg/ml)	3.1 ± 0.3	1.8 ± 0.7	0.0001
fT4 (0.34-5.60 mIU/L)	0.9 ± 0.2	0.5 ± 0.1	0.0001
TSH (0.34-5.60 mIU/L)	3.4 ± 1.3	8.2 ± 2.4	0.0001
hsCRP (<5 mg/L)	1.9 ± 1.2	8.5 ± 2.7	0.0001
LH (0.5-10.5 mIU/L)	5.8 ± 1.7	8.1 ± 3.0	0.0001
FSH (3.0-13.0 mIU/L)	6.3 ± 1.9	4.2 ± 1.6	0.0001
LH/FSH (<1.2)	0.9 ± 0.2	2.0 ± 0.8	0.0001
Prolactin (1.2-19.5 ng/ml)	13.5 ± 3.5	50.7 ± 27.1	0.0001


TC; Total cholesterol, TG; Triglycerides, HDL-c; High-density lipoprotein cholesterol, LDL-c; Low-density lipoprotein cholesterol, VLDL; Very low density lipoprotein, HOMA-IR; Homeostatic model assessment-insulin resistance, fT3; Free T3, fT4; Free T4, TSH; Thyroid stimulating hormone, hsCRP; High sensitive C-reactive protein, LH; Luteinizing hormone and FSH; Follicle stimulating hormone.

**Table 2:** Pearson correlation values for different parameters among polycystic ovary syndrome (PCOS) women


	BMI	hsCRP	Fasting glucose	Fasting insulin	HOMA-IR	TSH	LH	FSH	LH/FSH	Prolactin

BMI	1	0.158*	0.203*	0.439**	0.401**	0.173*	-0.279**	-0.207**	0.040	0.272**
hsCRP		1	0.816**	0.518**	0.609**	0.757**	0.279**	-0.514**	0.631**	0.688**
Fasting glucose			1	0.703**	0.811**	0.772**	0.256**	-0.533**	0.615**	0.699**
Fasting insulin				1	0.981**	0.499**	0.021	-0.410**	0.409**	0.542**
HOMA- IR					1	0.583**	0.049	-0.469**	0.466**	0.603**
TSH						1	0.222**	-0.440**	0.492**	0.693**
LH							1	0.244**	0.556**	0.089
FSH								1	-0.571**	-0.481**
LH/FSH									1	0.469**
Prolactin										1


*; Correlation is significant at the 0.05 level (2-tailed), **; Correlation is significant at the 0.01 level (2-tailed), BMI; Body mass index, hsCRP; High sensitive C-reactive protein, HOMA-IR; Homeostatic model assessment-insulin resistance, TSH; Thyroid stimulating hormone, LH; Luteinizing hormone and FSH; Follicle stimulating hormone.

## Discussion

PCOS is multi-factorial endocrine disorder associated with derangement in the metabolic profile and endocrine pattern. In the present study we have attempted to explore the changes in metabolic and hormonal parameters in PCOS women from Nellore and its surrounding districts of Andhra Pradesh, India. Out of 80 PCOS women recruited for the study, 42 women were in the age range of 20 to 25 years, 29 women were 25 to 30 years of age, and 9 women were 30 to 35 years of age. There were 15 patients who presented with a BMI lower than 20 kg/m^2^, 10 patients showed a normal BMI (20 to 25 kg/m^2^), 29 patients were overweight (BMI 25 to 30 kg/m^2^), and 26 patients were obese (BMI>30 kg/m^2^). The most common abnormalities seen in PCOS are increased BMI, low HDLc levels and elevated TG. In the present study an abnormal lipid profile was found in women with PCOS. The findings of elevated TC, TG, LDL-c and HDL-c agreed with those of Naidu et al. (2), Zhang et al. (16), Kim and Choi (17), Talbott et al. (18), and Saha et al. (19).

PCOS may represent (20) a major segment of the female population at a risk of cardiovascular disease, which may be related to increased VLDL-c levels. As shown by Wetterau et al. (21), this increase in VLDL-c is basically due to insulin resistance. Insulin normally inhibits the expression of microsomal triglyceride transfer protein that is responsible for apo-B and VLDL secretion. Hence, insulin resistance may be responsible for increased VLDL secretion in PCOS individuals (22). In the present study we have observed that 70% of PCOS patients exhibited an abnormal lipid profile and the mean values of cholesterol, TG, LDL-c and, VLDL-c were increased.

Hypothyroidism is the disease state caused by insufficient production of thyroid hormone by the thyroid gland. Some authors have affirmed that insulin resistance and increased androgen production can cause hypothyroidism. Insulin resistance has also been considered to be the principal factor in the genesis of PCOS (23). In our study, we observed an increased level of serum TSH and decreased level of fT3 and fT4 hormones. There were 29 out of 80 PCOS patients with TSH levels <5.5 mIU/L, 37 patients reported TSH levels in the range of 5.5 to 10 mIU/L, and 14 patients presented with TSH levels >10 mIU/L. The minimum TSH reported value was 3.6 and the maximum value was 13.4 mIU/L. fT3 and fT4 levels decreased (P˂0.0001) in PCOS women compared to controls. Our study results agreed with previous studies by Eldar-Geva et al. (24), Yasmin et al. (25), and Anwary et al. (26).

Insulin resistance and hyperinsulinemia are factors that play an important role in the pathogenesis of PCOS. In the present study we have shown predominant insulin resistance, hypothyroidism, dyslipidemia, and an increased LH/FSH ratio in women with POCS compared to control women (2, 5, 6). The direct effect of testosterone adipocytes has been investigated and induction of androgen receptor mediated insulin resistance via testosterone was established (27). Hyperandrogenism is due to increased LH and low-to-normal FSH levels. Due to the increase in LH and estrogen, FSH is negatively inhibited. Theca cell hyperplasia ensues, leading to hyperandrogenemia that clinically presents as hirsutism. In our study, we have used hirsutism as one of the clinical features for the diagnosis of hyperandrogenism. BMI has a negative association with the baseline levels of LH in PCOS patients. We observed this association in the present study, which supported results from previous studies (27, 28).

PCOS has been the subject of continuous studies on diagnosis, management, and therapy. During the 1980s-1990s, the LH/FSH ratio was perceived to be the gold standard for the diagnosis of PCOS. A higher LH/FSH ratio is no longer a characteristic attribute in PCOS as there is excess production of LH in PCOS patients, which is associated with the inconsistency in LH/FSH ratios in various studies. Of the 80 PCOS patients in this study, 18 patients presented with LH/FSH ratios lower than 1.5, 33 patients ranged from 1.5 to 2.0, and the remaining 29 had LH/FSH ratios higher than 2. Normal prolactin levels were reported in 15 patients, prolactin levels in the range of 19.5 to 50 ng/ml were shown in 26 patients, 36 patients had levels in the range of 51 to 100 ng/ml, and the remaining 3 patients presented with levels higher than 100 ng/ml.

In the current study, we observed a mild increase in the prolactin level (50.65 ± 27.11 ng/ml) in 68% cases, which was similar to previous studies conducted by Kalsum and Jalali (29) where 69.51% of subfertile women suffered from hyperprolactinemia. Nizam et al. (30) also showed that hyperprolactinemia was a major cause of subfertility. Treatment with drugs that lowered prolactin levels resulted in pregnancy for 24% of the infertile women. This finding was consistent with our study.

## Conclusion

In the present study, we showed the biochemical and hormonal imbalances that underlie the complex endocrinological cascade of PCOS in Nellore and its surrounding districts of the Andhra Pradesh population. PCOS patients in this population presented with a higher prevalence of insulin resistance, dyslipidemia, and hypothyroidism. Metabolic and endocrine patterns indicated that PCOS patients were at higher risk of developing diabetes as well as cardiovascular disease. An increased concentration of LH, mild hyperprolactinaemia, higher LH/FSH ratio, and decreased FSH has suggested that there is a disturbance in the normal gonadotropin ovarian axis. Further correlation study has revealed that LH is inversely proportional to BMI. A LH/FSH ratio greater than 2.0 can be useful in the diagnosis of PCOS women in our population. Hence we recommend that screening of PCOS patients for metabolic and endocrine parameters will help in the management and treatment of PCOS for a better outcome.
